# CYP2J2 Overexpression Protects against Arrhythmia Susceptibility in Cardiac Hypertrophy

**DOI:** 10.1371/journal.pone.0073490

**Published:** 2013-08-30

**Authors:** Christina Westphal, Bastian Spallek, Anne Konkel, Lajos Marko, Fatimunnisa Qadri, Laura M. DeGraff, Carola Schubert, J. Alyce Bradbury, Vera Regitz-Zagrosek, John R. Falck, Darryl C. Zeldin, Dominik N. Müller, Wolf-Hagen Schunck, Robert Fischer

**Affiliations:** 1 Max-Delbrueck Center for Molecular Medicine, Berlin, Germany; 2 Experimental and Clinical Research Center, a joint cooperation between the Charité Universitätsmedizin and the MDC, Berlin, Germany; 3 National Institute of Environmental Health Sciences, NIH, Research Triangle Park, North Carolina, United States of America; 4 Institute of Gender in Medicine, Charité Universitätsmedizin Berlin, Berlin, Germany; 5 University of Texas Southwestern Medical Center, Dallas, United States of America; 6 Department of Experimental Medicine I, Nikolaus-Fiebiger-Center for Molecular Medicine, Friedrich-Alexander-University Erlangen-Nürnberg, Germany; 7 Clinic for Cardiology and Pulmonology, Charité Universitätsmedizin Berlin, Berlin, Germany; Maastricht University Faculty of Health, Medicine, and Life Sciences, The Netherlands

## Abstract

Maladaptive cardiac hypertrophy predisposes one to arrhythmia and sudden death. Cytochrome P450 (CYP)-derived epoxyeicosatrienoic acids (EETs) promote anti-inflammatory and antiapoptotic mechanisms, and are involved in the regulation of cardiac Ca^2+^-, K^+^- and Na^+^-channels. To test the hypothesis that enhanced cardiac EET biosynthesis counteracts hypertrophy-induced electrical remodeling, male transgenic mice with cardiomyocyte-specific overexpression of the human epoxygenase CYP2J2 (CYP2J2-TG) and wildtype littermates (WT) were subjected to chronic pressure overload (transverse aortic constriction, TAC) or β-adrenergic stimulation (isoproterenol infusion, ISO). TAC caused progressive mortality that was higher in WT (42% over 8 weeks after TAC), compared to CYP2J2-TG mice (6%). In vivo electrophysiological studies, 4 weeks after TAC, revealed high ventricular tachyarrhythmia inducibility in WT (47% of the stimulation protocols), but not in CYP2J2-TG mice (0%). CYP2J2 overexpression also enhanced ventricular refractoriness and protected against TAC-induced QRS prolongation and delocalization of left ventricular connexin-43. ISO for 14 days induced high vulnerability for atrial fibrillation in WT mice (54%) that was reduced in CYP-TG mice (17%). CYP2J2 overexpression also protected against ISO-induced reduction of atrial refractoriness and development of atrial fibrosis. In contrast to these profound effects on electrical remodeling, CYP2J2 overexpression only moderately reduced TAC-induced cardiac hypertrophy and did not affect the hypertrophic response to β-adrenergic stimulation. These results demonstrate that enhanced cardiac EET biosynthesis protects against electrical remodeling, ventricular tachyarrhythmia, and atrial fibrillation susceptibility during maladaptive cardiac hypertrophy.

## Introduction

Cytochrome P450 (CYP)-dependent eicosanoids, such as epoxyeicosatrienoic acids (EETs) and 20-hydroxyeicosatetraenoic acid (20-HETE), may play crucial roles in the development of heart disease. EETs exert anti-inflammatory and antiapoptotic effects in cardiomyocytes and ameliorate cardiac ischemia-reperfusion injury, whereas 20-HETE causes detrimental effects in the same settings [1]–[4]. EETs also modulate the electrophysiological properties of the heart by regulating L-type Ca^2+^, Na^+^, and ATP-sensitive K^+^ (K_ATP_) channel activities [5]–[9]. In isolated hearts, exogenous EET administration improved postischemic functional recovery and prevented electrocardiogram abnormalities in the reperfusion phase [10], [11]. EET pretreatments also efficiently reduced myocardial infarction size after transient coronary artery occlusion [2], [12], [13]. Further studies revealed an essential role of EETs in mediating the beneficial effects of pre- and postconditioning [14]–[16].

CYP2J2 is the predominant arachidonic acid epoxygenase in the human heart [17]. CYP2J2-transgenic mice have been generated as a tool for investigating the impact of increased endogenous EET biosynthesis on cardiac disease development. The transgene contains the full-length CYP2J2 cDNA under control of the αMHC promoter and thus mediates cardiomyocyte-specific overexpression of the enzyme [18]. CYP2J2 overexpression reduced infarct size and improved recovery of pump function as well as ventricular repolarization after ischemia, thereby mimicking the effects of exogenous EET administration [18], [19].

We used CYP2J2-transgenic mice to test the hypothesis that enhanced cardiac EET biosynthesis prevents arrhythmogenic substrate formation during the development of maladaptive cardiac hypertrophy. We induced left ventricular hypertrophy by chronic pressure overload to predispose the mice to ventricular tachyarrhythmia and sudden cardiac death. Alternatively, we applied long-term isoproterenol infusion to mimic chronic β-adrenergic stimulation-induced cardiac hypertrophy and thereby established a model of atrial fibrillation susceptibility. We found that CYP2J2 overexpression mediated strong antiarrhythmic effects in both models, suggesting that EETs are involved in endogenous mechanisms preventing maladaptive electrical remodeling during cardiac hypertrophy.

## Results

### Effect of CYP2J2-overexpression on pressure overload-induced cardiac hypertrophy

**Figure 1:**
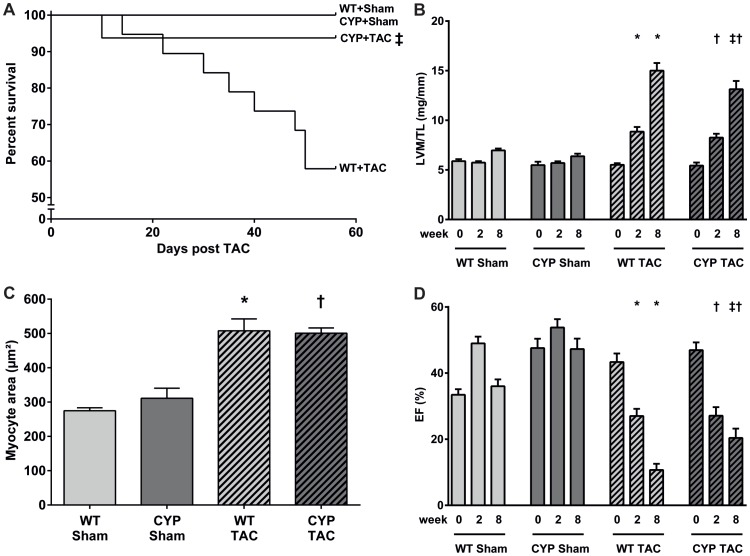
Chronic pressure overload-induced mortality and cardiac hypertrophy. (A) The survival rate was significantly higher in CYP2J2-TG (15 out of 16 animals survived over 8 weeks after TAC operation) compared with WT mice (11/19); Log rank-test ^‡^p<0.05. None of the sham operated WT (n = 17) or CYP2J2-TG mice (n = 11) died over the same period. (B) TAC-induced left ventricular hypertrophy was gradually ameliorated in CYP2J2-TG compared with WT mice. The difference was significant 8 weeks after TAC (13.1±0.8 vs. 15.1±0.8 mg/mm in 15 CYP2J2-TG vs. 11 WT mice). (C) Myocyte area significantly increased in both WT and CYP2J2-TG mice. (D) Systolic function was significantly decreased in both animal groups two weeks after TAC compared to the sham controls. Eight weeks after TAC, CYP2J2-TG mice (n = 15) showed significantly higher EF values (20.4±2.8 vs. 10.7±1.9%) than WT littermates (n = 11). Results represent mean±SEM; ANOVA, Post-Hoc Tukey; *p<0.05 vs. WT+Sham; ^†^p<0.05 vs. CYP+Sham; ^‡^p<0.05 vs. WT+TAC.

The survival rate during the development of pressure overload-induced cardiac hypertrophy was significantly higher in CYP2J2-TG compared to WT mice (Fig. 1A). In WT mice, progressive mortality started in week 3 after TAC operation. Only 11 out of 19 WT mice (58%) survived over 8 weeks. In contrast, 94% of the animals (15 out of 16) survived in the CYP2J2-TG group. After sham surgery, none of the WT (0/17) or CYP2J2-TG mice (0/11) died over the same post-operational period (Fig. 1A).

TAC induced a strong hypertrophic response (Figs. 1B and 1C) and severe decline of systolic function (Fig. 1D) in both animal groups. However, the development of these features was gradually ameliorated in CYP2J2-TG mice. Eight weeks after TAC, the left ventricular mass to tibia length-ratio (LVM/TL; Fig. 1B) was significantly lower (13.1±0.8 vs. 15.1±0.8 mg/mm) and the EF (Fig. 1D) better preserved (20.4±2.8 vs. 10.7±1.9%) in CYP2J2-TG compared to WT mice (compare Table S2 for the full set of echocardiographic data). CYP2J2-overexpression also moderately reduced the left ventricular expression of markers of hypertrophy (ANP and BNP) and fibrosis (Col1 and Col3); however, these effects as well as changes in the upregulation of βMHC were not statistically significant (Figs. S1A–E).

### Effect of CYP2J2-overexpression on pressure overload-induced electrical remodeling

Electrophysiological studies were performed 4 weeks after TAC. This time point was selected to identify features of electrical remodeling potentially related to the onset of increased mortality in WT and improved survival of CYP2J2-TG mice (compare Fig. 1A). TAC-induced cardiac hypertrophy was associated with significant QRS prolongation in WT but not CYP2J2-TG mice (Table 1). The ventricular effective refractory period (VERP) increased in both animal groups after TAC; however, this effect was significantly more pronounced in CYP2J2-TG compared with WT mice (Table 1). Four weeks after TAC, the VERP values of CYP2J2-TG hearts exceeded those of WT hearts by almost 15 ms.

**Table 1:** Electrophysiological parameters of WT and CYP2J2-TG mice four weeks after TAC or sham operation.

	WT Sham	CYP Sham	WT TAC	CYP TAC
**HR (bpm)**	459.2±7.2	483.6±17.4	599.3±20.4*	**511.8±27.6** ‡
**P (ms)**	16.7±0.2	16.5±0.2	14.4±0.4*	15.8±0.6
**PR (ms)**	39.4±1.1	38.8±0.7	42.0±5.5	39.8±1.0
**QRS (ms)**	10.9±0.3	11.7±0.3	14.0±1.4*	12.0±0.3
**QTc (ms)**	53.7±1.3	58.6±1.0	55.3±2.0	54.6±1.7
**AV WB (ms)**	73.1±3.4	73.7±1.6	69.6±1.9	69.2±2.4
**AV 2∶1 (ms)**	52.0±1.9	52.0±1.0	53.6±1.2	50.4±2.7
**AVNERP (ms)**	50.2±1.6	48.0±1.0	44.3±1.8	45.6±0.8
**AERP (ms)**	20.3±1.0	14.7±0.6	16.7±1.4	18.4±1.4
**VERP (ms)**	24.0±1.4	31.4±0.8*	30.9±2.2*	45.6±1.2*† ‡

WT – Wildtype; CYP – CYP2J2 overexpressing mice; TAC – Transverse aortic constriction; HR – Heart rate; bpm – Beats per minute; ms – milliseconds; P – P-wave duration; PR – PR interval; QRS – QRS interval; QTc – QT interval (corrected for heart rate); AV WB – 1∶1 Atrioventricular node conduction capacity ( = Wenckebach point); AV 2∶1 - 2∶1 Atrioventricular node conduction capacity; AVNERP – Atrioventricular node effective refractory period; AERP – Atrial effective refractory period; VERP - Ventricular effective refractory period.

p<0.05 * vs. WT Sham,

† vs. CYP Sham,

‡ vs. WT TAC.

**Figure 2:**
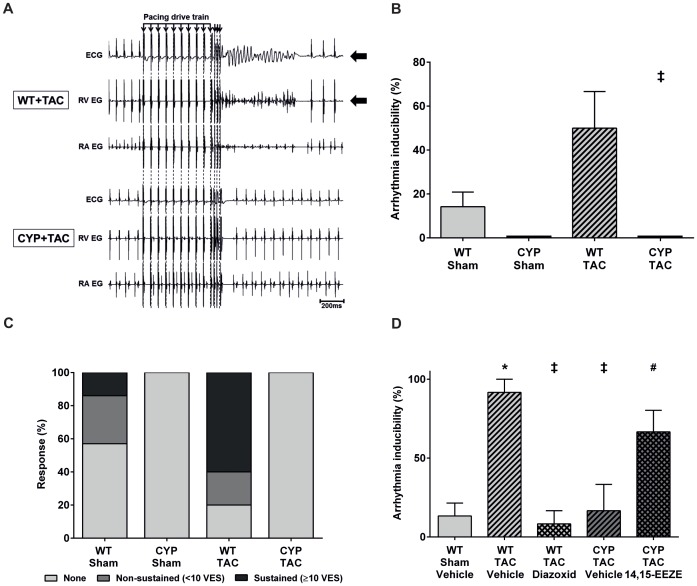
Arrhythmia susceptibility after pressure overload-induced cardiac hypertrophy. (A) Representative original tracings showing the induction of ventricular tachyarrhythmia by programmed electrical stimulation in WT mice 4 weeks after TAC (upper panel) and the resistance of TAC operated CYP2J2-TG mice under the same conditions (lower panel). (B) Ventricular arrhythmia inducibility significantly increased in WT mice after TAC (n = 5) compared with the sham control (n = 7). Arrhythmias were not inducible in any of the CYP2J2-TG mice both after sham (n = 5) and TAC operation (n = 6). Each animal was subjected to three protocols of programmed electrical stimulation and statistical evaluation was performed as described in Materials and Methods. (C) The severity of ventricular tachyarrhythmias scored according to the length of induced episodes (number of consecutive ventricular extrasystoles; VES) increased in WT mice after TAC compared with the sham control, whereas neither non-sustained nor sustained arrhythmias were inducible in corresponding CYP2J2-TG mice. (D) Analysis of arrhythmia inducibility in Langendorff preparations of isolated perfused hearts (n = 4 per group). Comparison of the vehicle treated groups confirmed the contrasting vulnerabilities of hypertrophied WT and CYP2J2-TG hearts after TAC. Perfusion with the mitochondrial K_ATP_-channel opener diazoxide (100 µM, 20 min prior to programmed electrical stimulation) reduced the arrhythmia inducibility of WT-TAC hearts to the levels of hearts isolated from sham WT mice as well as CYP2J2-TG mice after TAC. Pretreatment with the EET antagonist 14,15-EEZE-mSi (48.5 µM for 20 min) reversed the protection of hypertrophied CYP2J2-TG hearts towards arrhythmia inducibility. Results represent mean±SEM; ANOVA, Post-Hoc Tukey; *p<0.05 vs. WT-Sham (vehicle); ^‡^p<0.05 vs. WT-TAC (vehicle); # p<0.05 vs. CYP-TAC (vehicle).

In WT, but not CYP2J2-TG mice, chronic pressure overload significantly increased the vulnerability to ventricular tachyarrhythmia (Fig. 2A). In WT-TAC mice, 47% (7/15) of the stimulation protocols were effective in contrast to only 14% (3/21) in the WT-sham group (Fig. 2B). Sustained arrhythmic episodes lasting longer than 10 consecutive VES predominated in WT-TAC mice. The sham controls showed mostly either no or only non-sustained arrhythmias (Fig. 2C). In contrast, CYP2J2-TG mice were completely resistant. The same PES protocols that were effective in WT mice did not induce cardiac arrhythmias in any of the CYP2J2-TG animals 4 weeks after sham or TAC operation (Fig. 2).

Consistent with the *in vivo* results, large differences in ventricular arrhythmia susceptibility were also detectable in Langendorff preparations of perfused hearts isolated from WT and CYP2J2-TG mice 4 weeks after TAC (Fig. 2D). Epicardial electrical stimulation induced ventricular arrhythmias in more than 90% of the protocols with the hypertrophied WT hearts compared with only 15% with the corresponding CYP2J2-TG hearts. The high arrhythmia inducibility of WT-TAC hearts was strongly reduced after perfusing the organs with the mitochondrial K_ATP_-channel opener diazoxide for 20 min prior to stimulations (Fig. 2D). Conversely, the selective EET-antagonist 14,15-EEZE-mSi reversed the protection of hypertrophied CYP2J2-TG hearts against ventricular arrhythmia inducibility (Fig. 2D).

**Figure 3:**
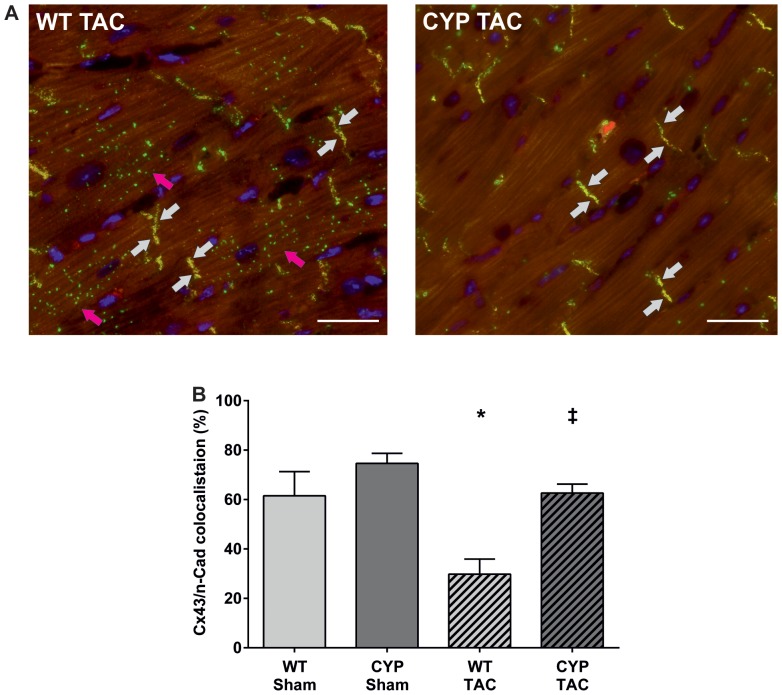
Chronic pressure overload-induced alterations in Cx43 localization. (A) Representative immunofluorescence staining of left ventricular cryosections prepared from WT and CYP2J2-TG mice 4 weeks after TAC surgery. The sections were co-stained for detecting Cx43 (green fluorescent signal) and N-cadherin (red). Cx43 and N-cadherin are colocalized (yellow) to the intercalated disks (indicated by white arrows). This normal Cx43 localization was largely preserved in CYP2J2-TG mice, whereas WT mice featured TAC- induced redistribution of Cx43 to the cytoplasm and lateral borders of the cardiomyocytes (pink arrows). Nuclei were stained with DAPI (blue). Scale bar: 50 µm. (B) Quantitative analysis of Cx43 and N-cadherin colocalization. Results represent mean±SEM based on the analysis of 5 sections per heart and 4–6 animals per group; ANOVA, Post-Hoc Tukey; *p<0.05 vs. WT-Sham; ^‡^p<0.05 vs. WT-TAC.

Since gap junctional remodeling may predispose to arrhythmia, we also analyzed the left ventricles for TAC-induced changes in the intracellular localization of connexin 43 (Cx43), the major ventricular gap junction protein (Fig. 3). Double immunostaining with antibodies directed against Cx43 and N-cadherin revealed a redistribution of Cx43 from the intercalated discs to the cytoplasm or lateral boarders in WT mice 4 weeks after TAC. In contrast, the left ventricles of CYP2J2-TG mice were largely protected against gap junctional remodeling upon chronic pressure overload. Typically, the Cx43 expression was preserved in intercalated discs with only little redistribution (Fig. 3).

### Effect of CYP2J2-overexpression on chronic β-adrenergic stimulation-induced cardiac hypertrophy

**Figure 4:**
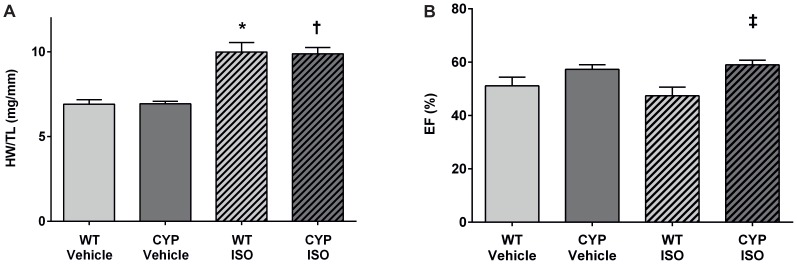
Induction of cardiac hypertrophy by chronic β-adrenergic stimulation. (A) Two weeks of chronic ISO infusion significantly increased the heart weight to tibia length-ratio in WT and CYP2J2-TG mice (n = 7 per group) compared with the vehicle controls (n = 7 and 5). The hypertrophic response was not different in CYP2J2-TG compared to WT mice. (B) Systolic function was not significantly altered upon chronic ISO infusion as indicated by preserved EF values compared to the respective vehicle controls. EF was slightly but significantly higher in CYP2J2-TG than WT mice two weeks after chronic ISO stimulation. Results represent mean±SEM; ANOVA, Post-Hoc Tukey; *p<0.05 vs. WT+Vehicle; †p<0.05 vs. CYP+Vehicle; ^‡^p<0.05 vs. WT+ISO.

Chronic ISO infusion caused moderate cardiac hypertrophy in both WT and CYP2J2-TG mice (Fig. 4A). After two weeks, the heart weight to tibia length ratio (HW/TL) was significantly higher in ISO compared with vehicle treated animals. However, the hypertrophic response was not different comparing WT and CYP2J2-TG mice (Fig. 4A). Also, the ISO-induced increases in heart rate were almost identical in WT and CYP2J2-TG mice (Table 2). Systolic function (EF) was significantly higher in CYP2J2-TG compared with WT mice (59±2 vs. 47±3%) two weeks after ISO but not vehicle treatment (Fig. 4B; compare Table S3 for the full set of echocardiographic data).

**Table 2:** Electrophysiological parameters of WT and CYP2J2-TG mice two weeks after chronic vehicle or isoproterenol infusion.

	WT Vehicle	CYP Vehicle	WT ISO	CYP ISO
**HR (bpm)**	509.0±14.7	507.8±39.5	650.7±3.9*	**642.9±7.4** †
**P (ms)**	16.1±0.4	15.4±0.2	15.8±0.4	16.8±0.2
**PQ (ms)**	38.8±0.9	39.2±1.0	35.3±0.6*	**34.5±0.8** †
**QRS (ms)**	10.5±0.3	10.8±0.4	9.6±0.2	10.5±0.2
**QTc (ms)**	53.1±1.1	52.6±0.8	49.7±0.9	51.4±1.0
**AV WB (ms)**	71.3±1.3	67.5±0.5	67.8±1.0	70.0±1.8
**AV 2∶1 (ms)**	52.0±0.5	50±1.6	49.6±0.6	53.3±1.4
**AVNERP (ms)**	48.7±1.3	46.3±1.1	46.7±1.2	**48.7±1.3** ‡
**AERP (ms)**	21.7±0.9	22.2±1.1	13.1±0.5*	**18.0±0.7** † ‡
**VERP (ms)**	28.9±1.0	36.1±1.9	30.5±0.6	36.8±0.6

WT – Wildtype; CYP – CYP2J2 overexpressing mice; TAC – Transverse aortic constriction; HR – Heart rate; bpm – Beats per minute; ms – milliseconds; P – P-wave duration; PR – PR interval; QRS – QRS interval; QTc – QT interval (corrected for heart rate); AV WB – 1∶1 Atrioventricular node conduction capacity ( = Wenckebach point); AV 2∶1 - 2∶1 Atrioventricular node conduction capacity; AVNERP – Atrioventricular node effective refractory period; AERP – Atrial effective refractory period; VERP - Ventricular effective refractory period.

p<0.05 * vs. WT Vehicle.

† vs. CYP Vehicle.

‡ vs. WT ISO.

### Effect of CYP2J2-overexpression on chronic β-adrenergic stimulation-induced electrical remodeling

Chronic β-adrenergic stimulation specifically modulated the atrial effective refractory period (AERP) without having any detectable effect on ventricular refractoriness (Table 2). ISO-treatment strongly decreased the AERP in WT mice. This shortening of atrial refractoriness was significantly ameliorated in CYP2J2-TG mice.

**Figure 5:**
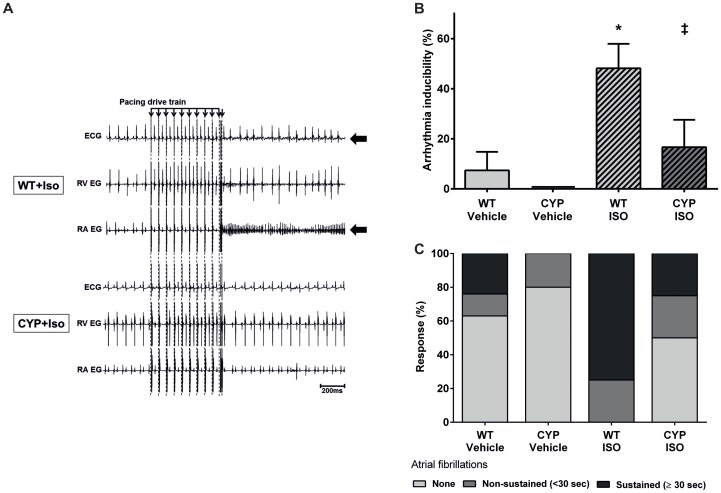
Arrhythmia susceptibility after chronic β-adrenergic stimulation-induced cardiac hypertrophy. (A) Representative original tracings showing the induction of atrial fibrillation by programmed electrical stimulation in WT mice 2 weeks after chronic ISO infusion (upper panel) and the resistance of CYP2J2-TG mice treated in the same manner (lower panel). (B) Atrial fibrillation inducibility significantly increased in WT mice after chronic ISO infusion (n = 9) compared with the vehicle control (n = 8) and was significantly higher than in CYP2J2-TG mice (n = 7 and n = 8 for the vehicle and ISO groups). (C) The relative percentage of inducible sustained atrial fibrillation was markedly higher in WT compared with CYP2J2-TG after chronic ISO infusion. For statistical evaluation of arrhythmia inducibilities and severity scoring compare Fig. 2. Results represent mean±SEM; ANOVA, Post-Hoc Tukey; *p<0.05 vs. WT+Vehicle; †p<0.05 vs. CYP+Vehicle; ^‡^p<0.05 vs. WT+ISO.

In line with its specific effect on atrial refractoriness, ISO significantly increased the inducibility of atrial but not ventricular arrhythmia in WT mice (Fig. 5A). After ISO treatment, almost 50% of the PES protocols (13 out of 27 in 9 animals) induced atrial fibrillation in WT mice compared with only about 9% (2 out of 22 in 8 animals) in the vehicle control (Fig. 5B). We also observed a higher proportion of atrial fibrillation episodes lasting longer than 30 seconds, which we considered as sustained arrhythmias. The percentage of animals showing sustained atrial fibrillation in any of the PES protocols increased from about 30 to 80% comparing vehicle and ISO-treated WT mice (Fig. 5C).

In contrast to WT mice, CYP2J2-TG mice were largely protected against the development of atrial fibrillation inducibility. Two weeks after ISO treatment, CYP2J2-TG mice showed an atrial fibrillation inducibility of about 17% (4 out of 24 protocols in 8 animals) that was significantly lower than in the corresponding WT group (Fig. 5B). Also, the relative percentage of induced sustained fibrillations was markedly lower in CYP2J2-TG than in WT-mice (Fig. 5C).

Chronic β-adrenergic stimulation did not increase the vulnerability to ventricular arrhythmia. The ratios of effective vs. total protocols were 1/24 vs. 3/24 for vehicle and ISO treated WT mice, and 0/15 vs. 0/24 for vehicle and ISO treated CYP2J2-TG mice. The ECG parameters of vehicle-treated WT and CYP2J2-TG mice were not significantly different (Table 2). ISO treatment for two weeks clearly reduced PQ interval in both animal groups compared to the vehicle controls. Other ECG parameters remained essentially unchanged. In particular, ISO treatment did not induce QRS or QTc prolongation neither in WT nor CYP2J2-TG mice.

**Figure 6:**
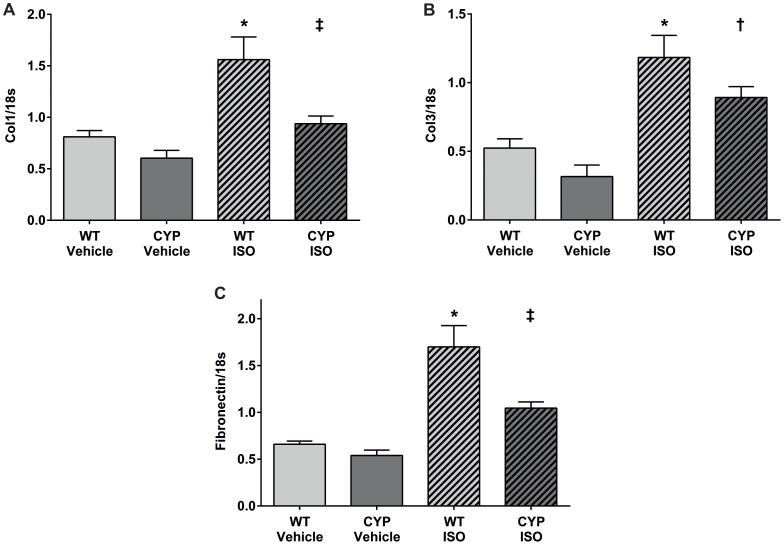
Effect of chronic β-adrenergic stimulation on the expression of markers of fibrosis in WT and CYP2J2-TG mice. RNA isolated from atrial tissue 2 weeks after vehicle or ISO infusion was reverse transcribed and analyzed by quantitative Taqman- or SYBR-PCR for the expression of Col1 (A), Col3 (B) and fibronectin (C). WT+Vehicle: n = 8; CYP+Vehicle n = 5; WT+ISO: n = 10; CYP+ISO: n = 8. ANOVA, Post-Hoc Tukey; *p<0.05 vs. WT+vehicle ^†^p<0.05 vs. CYP+vehicle; ^‡^p<0.05 vs. WT+ISO.

Chronic β-adrenergic stimulation resulted in the development of atrial fibrosis as indicated by significantly increased Col1, Col3 and fibronectin mRNA levels in ISO- compared to vehicle-treated WT mice (Figs. 6A–C) and confirmed by Sirius red staining of atrial sections (Fig. S2). The expression of these features of ISO-induced atrial fibrosis was clearly ameliorated in CYP2J2-TG mice (Figs. 6A–C and Fig. S2).

## Discussion

Our study shows that cardiomyocyte-specific overexpression of the human epoxygenase CYP2J2 protects against arrhythmia susceptibility in two mouse models of cardiac hypertrophy. CYP2J2-overexpression reduced the vulnerability towards ventricular tachyarrhythmia after chronic pressure overload (TAC model), and suppressed atrial fibrillation inducibility after chronic β-adrenergic stimulation (ISO model). The beneficial effects on cardiac electrical stability occurred without, or only moderately, reducing the hypertrophic response. Our data suggest that CYP2J2 overexpression prevented arrhythmogenic substrate formation primarily by maintaining gap junction integrity in the TAC model and by attenuating the development of atrial fibrosis in the ISO model. In isolated hypertrophied hearts, the EET antagonist 14,15-EEZE-mSi reversed the antiarrhythmic effect of CYP2J2 overexpression, indicating a direct role of CYP2J2-derived metabolites in preserving cardiac electrical stability.

Pressure overload-induced cardiac hypertrophy is associated with structural and electrical remodeling eventually leading to heart failure and increased propensity to ventricular tachyarrhythmia and sudden cardiac death [20]. CYP2J2 overexpression markedly reduced the mortality after TAC. However, cardiac hypertrophy and decline in pump function were only moderately ameliorated. Thus, we assumed that the increased survival rate of CYP2J2-transgenic mice was predominantly related to an improvement of cardiac electrical stability. Supporting this notion, ventricular tachyarrhythmia inducibility that strongly increased in WT mice after TAC was completely suppressed in CYP2J2-TG mice. Moreover, CYP2J2 TG mice displayed less severe QRS interval prolongation than WT mice. QRS prolongation indicates ventricular conduction slowing and has been considered as a predictor of mortality in congestive heart failure patients [21], [22]. In agreement with the antiarrhythmic effect, CYP2J2 overexpression increased ventricular refractoriness. Prolongation of VERP protects against reentry tachycardias as known from the mechanism of action of antiarrhythmic drugs. EETs inhibit the open probability of cardiac Na^+^-channels resembling the action of Class I antiarrhythmics such as lidocaine [9]. This effect may have contributed to the ventricular refractoriness prolongation observed in CYP2J2-TG mice. Action potential prolongation, as induced by Class III antiarrhythmics, would provide an alternative explanation. However, in contrast to this expectation, cardiac action potential duration is significantly shortened in CYP2J2-TG compared with WT mice [7] and we did not observe QT interval prolongation.

TAC-induced cardiac hypertrophy was associated with gap junction remodeling as indicated by delocalization of Cx43 in WT mice. In contrast, the hypertrophied hearts of CYP2J2-TG mice showed a preserved Cx43 expression pattern. Reduced and heterogeneous Cx43 expression causes ventricular conduction slowing and irregular impulse propagation and thus increases the risk of fatal ventricular tachyarrhythmia [23], [24]. Changes in the phosphorylation state of Cx43 are obviously responsible for loss of gap junction integrity during chronic pressure overload. Providing an impressive proof of this notion, mice expressing a phosphatase-resistant mutant of Cx43 are protected against TAC-induced gap junctional remodeling and development of arrhythmia vulnerability [25]. Our study suggests that CYP2J2-derived metabolites such as EETs may play a critical role in the regulation of ventricular Cx43 remodeling. The mechanism may be related to the capacity of EETs to activate mitochondrial K_ATP_ channels. Mitochondrial K_ATP_ activity is higher in cardiomyocytes from CYP2J2-TG compared to WT mice and can be increased in WT cardiomyocytes by exogenous EET administration [18]. Activation of mitochondrial K_ATP_ channels by ischemic preconditioning or diazoxide protects against ischemia-induced Cx43 redistribution and electrical uncoupling [26]. Inhibition of mitochondrial K_ATP_ channels blunts arrhythmia protection in ischemic exercised hearts [27]. In our study, short-term diazoxide treatment abolished the high arrhythmia susceptibility of hypertrophied WT hearts, indicating that increased mitochondrial K_ATP_ channel activity would be indeed sufficient to confer the protection observed in CYP2J2-TG mice. Indicating a general link between EETs and connexins, EETs also increase inter-endothelial and myoendothelial gap junctional communication in the vasculature [28], [29]. However, further studies are necessary to understand the actual molecular mechanisms that may link EET-mediated mitochondrial K(ATP) channel activation to protection against Cx43 redistribution and arrhythmia.

Atrial fibrillation is the most common chronic cardiac arrhythmia in humans [30], [31]. Adrenergic stimulation from catecholamines can cause atrial fibrillation in patients [32]. By chronic ISO infusion in mice, we have established a new disease-relevant model for investigating the mechanisms of arrhythmogenic atrial remodeling. We observed, presumably for the first time, that chronic β-adrenergic stimulation indeed enhances atrial fibrillation inducibility without increasing the vulnerability to ventricular arrhythmia in WT mice. Consistent with the specificity of this effect, atrial but not ventricular refractoriness was decreased. CYP2J2-overexpression protected against AERP shortening and atrial fibrillation induction. However, CYP2J2 overexpression did not affect the general hypertrophic or chronotropic response to chronic ISO infusion. Arrhythmogenic atrial remodeling was associated with increased fibrosis in WT mice. This feature was significantly ameliorated in CYP2J2-TG mice. Atrial fibrosis causes conduction abnormalities and is generally considered as an important component of the remodeling process creating the substrate for atrial fibrillation [33].

Previous studies used inhibitors of the soluble epoxide hydrolase (sEH) or sEH knockout mice to increase the cardiovascular EET levels by preventing the metabolism of EETs to the corresponding less active dihydroxyeicosatrienoic acids [34]. These measures were highly effective in protecting against pressure overload- and angiotensin II-induced cardiac hypertrophy and heart failure. Some of these studies also showed that blockade of sEH protects against increased cardiac arrhythmia susceptibility [35], [36]. However, whether reduced arrhythmia vulnerability was a concomitant feature of reduced hypertrophy and heart failure or due to mechanisms that specifically ameliorate electrical remodeling remained unclear. Combined with the results of the present study, we can now conclude that the epoxy-metabolites produced by CYP2J2 or stabilized upon sEH inhibition exert a direct antiarrhythmic effect that protects the heart from arrhythmia even under conditions of severe hypertrophy and pump failure.

## Materials and Methods

Detailed Methods are provided in the Online Supplement.

### Animals

Male CYP2J2-transgenic mice (CYP-TG) and corresponding wild-type (WT) littermates [18] were kept on a 12 h/12 h light/dark cycle in temperature-controlled rooms and fed with standard chow (ssniff, Soest, Germany) and water *ad libitum*. All animal procedures were performed in accordance with the guidelines of the American Physiological Society and were approved by local authorities (Landesamt für Gesundheit und Soziales, Berlin, Germany).

### Pressure overload-induced cardiac hypertrophy

Transverse aortic constriction (TAC) was performed as described previously [37]. Left ventricular mass (LVM) and ejection fraction (EF) were determined by echocardiography following published procedures [38]. Cohort 1 was analyzed four weeks after TAC by ECG and electrophysiological studies *in vivo* or in an isolated heart system. Cohort 2 was sacrificed eight weeks after TAC or sham surgery.

### Cardiac hypertrophy upon chronic β-adrenergic stimulation

ISO was continuously infused via subcutaneously implanted osmotic minipumps (Alzet) at a rate of 40 mg/kg/d for two weeks. Echocardiography was performed before and after 13 days of ISO infusion. After two weeks of treatment, ECG and electrophysiological data were recorded.

### In vivo electrophysiological studies

Programmed electrical stimulation (PES) was performed in the right atrium or right ventricle using a digital electrophysiology lab (EP Tracer; CardioTek) to determine refractory periods and arrhythmia inducibility [39]. Atrial arrhythmias were defined as fast (>800 bpm) electrical activity in the right atrial electrograms, with ECG P waves different to normal sinus rhythm and subsequent fast, but physiological activation of the ventricles (ECG R wave and right ventricular electrograms similar to normal sinus rhythm). Atrial fibrillation was defined as fast, irregular activity in the right atrial electrograms with irregular conduction to the ventricles (high variability of R-R intervals). Ventricular arrhythmias were defined by fast (>800 bpm) activity originating from the ventricular myocardium (change in morphology of ECG R waves and local right ventricular electrograms compared to normal sinus rhythm). During inhalation anesthesia with isoflurane (2% with 360 ml/min air flow; Univentor 400 anesthesia unit), the animals' body temperature was kept constant at 37°C using a homeothermic blanket control unit (Hugo Sachs Elektronik, Harvard Apparatus) with rectal temperature control. After preparation of the right jugular vein, a 2 French octapolar electrophysiology catheter (CIB'ER mouse cath; NuMed) was placed in the right heart, including atrium and ventricle. PES was performed using a standardized protocol that included trains of 10 basal stimuli (S1) followed by up to 3 extra stimuli (S2–S4), delivered with a coupling interval decreasing in steps of 5 ms until ventricular or atrial refractoriness was reached. The stimulation procedures were repeated at three different basal cycle lengths (100 ms, 90 ms, 80 ms) with each animal. Occurrence and duration of inducible arrhythmias were documented. Only stimulation protocols with reproducible arrhythmias longer than five consecutive beats in ventricle and episodes longer than 350 ms in the atria were considered positive. “Arrhythmia inducibility” was calculated as the percentage of effective (positive) out of total protocols applied. Accordingly, the arrhythmia inducibility of individual animals could take a value of 0, 33, 66 or 100%. For statistical evaluation, the data obtained for the individual animals in a given group were averaged and are given as mean±SEM. For scoring the severity of induced arrhythmias, three response categories were defined: sustained (≥10 consecutive ventricular extrasystoles, VES or atrial fibrillation episodes ≥30 sec in at least one protocol), non-sustained (<10 VES or atrial fibrillation episodes <30 sec in at least one protocol) and no arrhythmias in all three protocols. The data are given as percentage of animals in a given group assigned to these categories.

### Electrocardiography (ECG)

Standard limb lead surface ECGs were recorded under slight isoflurane anesthesia using skin electrodes. Calculation of standard ECG time-interval parameters were performed by two independent operators based on averaged ECG lead II waveforms. Corrections for heart rate and definition of time points were made as described previously [39].

### Langendorff preparation of isolated hearts

The mice were injected i.p. with 40 I.U. of Heparin-Na in PBS and were sacrificed after 10 min. Hearts were rapidly excised and placed in ice-cold modified Krebs-Henseleit buffer, and the aortas were cannulated. The mounted hearts were perfused in a retrograde fashion at constant pressure (60 mm Hg) with continuously aerated (95% O_2_–5% CO_2_) modified Krebs-Henseleit buffer containing (in mmol/l) 118 NaCl, 4.7 KCl, 1.2 MgSO_4_, 1.5 CaCl_2_, 24.7 NaHCO_3_, 0.23 KH_2_PO_4_, 0.06 EDTA and 11.1 glucose, at a temperature of 37°C. The hearts were stabilized for 10 min and then treated for 20 min with either the mitochondrial K_ATP_-channel opener diazoxide (100 µmol/l in the perfusion buffer), a selective EET-antagonist (48.5 µmol/l 14,15-epoxyeicosa-5(Z)-enoic-methylsulfonylimide; 14,15-EEZE-mSI [40]) or as control with vehicle (0.1% dimethylsulfoxide; DMSO). A pacing electrode was placed at the left ventricle and the same PES protocols to determine ventricular refractoriness and inducibility of ventricular arrhythmias were performed as used *in vivo*.

### Histology

In the TAC model, frozen tissue was embedded in O.C.T-Tissue Tek (Sakura, Netherland) and cut in 2 µm sections. Connexin 43 (Cx43) was stained using anti-Cx43 antibody (1∶1000; from Sigma-Aldrich) and Alexa488-conjugated secondary anti-rabbit antibody (1∶500; Jackson-Immuno Research). Gap junction protein N-cadherin was stained using anti-N-cadherin antibody (3 µg/ml; from Invitrogen) and secondary Cy3-conjugated secondary anti-mouse antibody (1∶300; Dianova). Cx43 location was quantified by determining the colocalization of Cx43 and N-cadherin in the intercalated discs as described before [25].

Myocyte area was measured from cross-sectional area of cardiomyocytes stained by wheat germ agglutinin (WGA) labeled with Oregon Green 488 (1∶500; Life Technologies).

### Gene expression

mRNA levels of atrial and brain natriuretic peptides (ANP and BNP) βmyosin heavy chain (βMHC), collagen 1 and 3 (Col1 and Col3), and fibronectin were determined by Taqman or SYBR RT-PCR protocols using 18S RNA as endogenous control. The primers used are given in Table S1.

### Data analysis and statistics

All data were tested for normal distribution and are given as mean±SEM. Data were analyzed by a two-way ANOVA and post hoc Tukey's test using SPSS17 for Windows. Survival differences were analyzed by the log-rank test. P-values <0.05 were defined as statistically significant.

## Supporting Information

Materials S1 **Supplementary material.**
(DOC)

Figure S1 **Effect of chronic pressure overload on the expression of markers of hypertrophy and fibrosis in WT and CYP2J2-TG mice.**
(DOCX)

Figure S2 **Effect of chronic β-adrenergic stimulation on atrial fibrosis in WT and CYP2J2-TG mice.**
(DOCX)

Table S1 Primer sequences for quantitative real-timeRT-PCR.
(DOCX)

Table S2 Summary of echocardiographic data eight weeks after TAC.
(DOCX)

Table S3 Summary of echocardiographic data after two weeks of β-adrenergic stimulation.
(DOCX)
