# Supplementary material for: CYP2J2 Overexpression Protects against Arrhythmia Susceptibility in Cardiac Hypertrophy
Source: PLoS One. 2013 Aug 30;8(8):e73490. doi: 10.1371/journal.pone.0073490 (PMC3758319; doi:10.1371/journal.pone.0073490)
Supplement: Materials S1 — Supplementary material. (DOC) [file pone.0073490.s001.doc]

**Supplementary Material**

**Westphal et al. CYP2J2 overexpression protects against arrhythmia susceptibility in cardiac hypertrophy**

*Transverse aortic constriction model*

The mice were anesthetized by intraperitoneal (IP) injection of 1mg/kg Ketamine (80 mg/ml) in combination with Xylazine (12 mg/ml; Sigma Aldrich). After the thorax was opened, banding was performed between the truncus brachiocephalicus and the left common carotid artery. Ligature was made with a 6-0 silk suture and the help of a 26-gauche needle to ensure a standardized diameter of 0.4mm. The thorax was then closed and the animals recovered under red light. Sham operated animals underwent the same procedure without the ligation of aorta. All animals got an analgetic treatment with Rimadyl® (Pfizer; 4.0mg/kg BW; IP).

*Echocardiographic measurements*

All echocardiographic procedures were as described previously.^1^ Left ventricular mass (LVM) and ejection fraction (EF) was calculated from the short axis view, whereas the pressure gradient was calculated from ascending and descending aorta.

*Gene expression*

Total RNA was isolated from snap-frozen heart tissues using the RNeasy RNA isolation kit (Qiagen, Germany) according to the manufacturer's instruction. Isolated RNA concentration was measured and RNA quality was tested by NanoDrop-1000 spectrophotometer (PeqLab, Germany). For the synthesis of cDNA, equivalent amounts of RNA (2 μg) were used and was performed by a high capacity cDNA reverse transcription kit (Applied Biosystems, Germany).

Quantitative analysis of target mRNA expression was performed with real-time RT-PCR using the relative standard curve method. TaqMan and SYBR green analysis was conducted according to the manufacturer’s instructions, using an Applied Biosystems 7500 Sequence Detector (Applied Biosystems, Germany). The expression level of the target genes was normalized by 18s. Primers were synthesized by Biotez (Berlin, Germany) and listed in Table 1.

*References*

1. *Quinones MA, Otto CM, Stoddard M, Waggoner A, Zoghbi WA. Recommendations for quantification of doppler echocardiography: A report from the doppler quantification task force of the nomenclature and standards committee of the american society of echocardiography. J Am Soc Echocardiogr. 2002;15:167-184*
