# Supplementary material for: CYP2J2 Overexpression Protects against Arrhythmia Susceptibility in Cardiac Hypertrophy
Source: PLoS One. 2013 Aug 30;8(8):e73490. doi: 10.1371/journal.pone.0073490 (PMC3758319; doi:10.1371/journal.pone.0073490)
Supplement: Figure S1 — Effect of chronic pressure overload on the expression of markers of hypertrophy and fibrosis in WT and CYP2J2-TG mice. (DOCX) [file pone.0073490.s002.docx]

**Figure S1A**

**
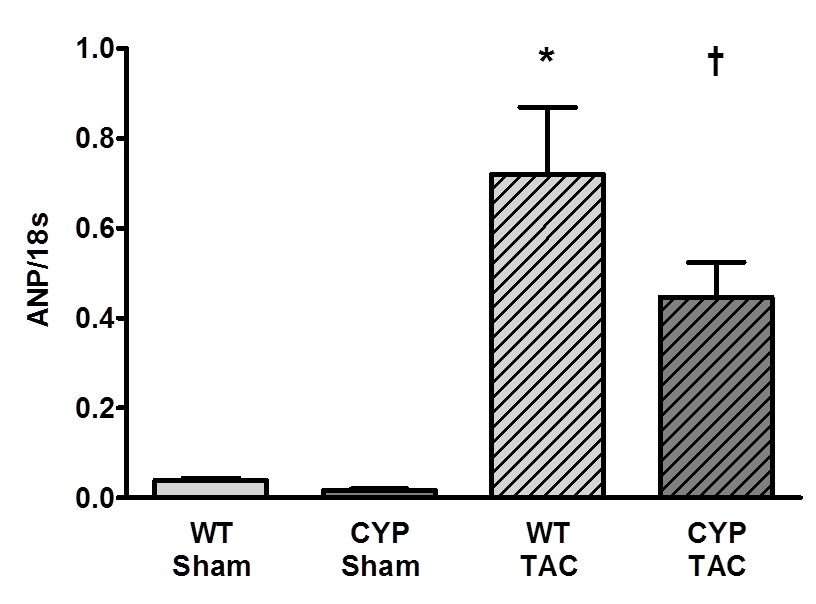
**

**Figure S1B**


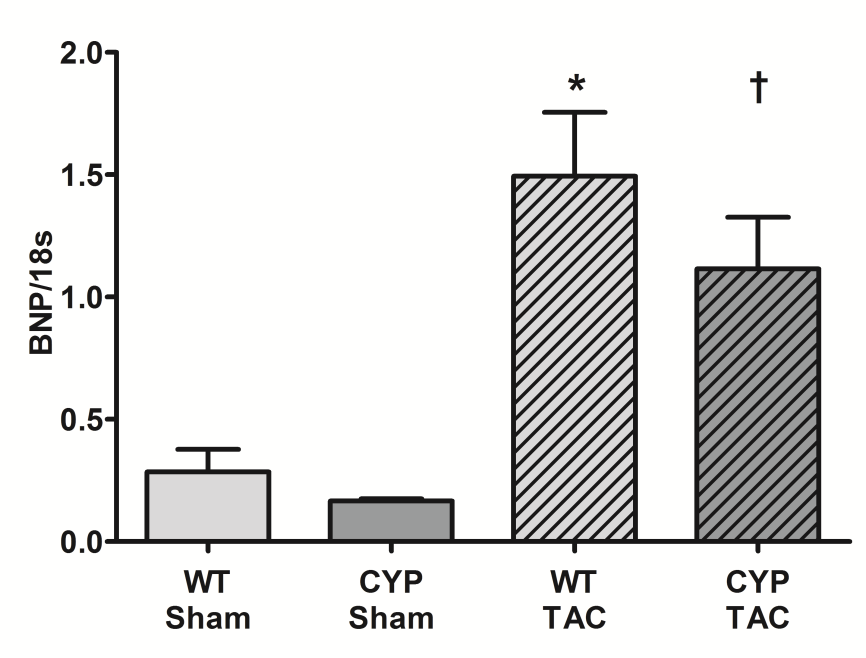


**Figure S1C:**

**
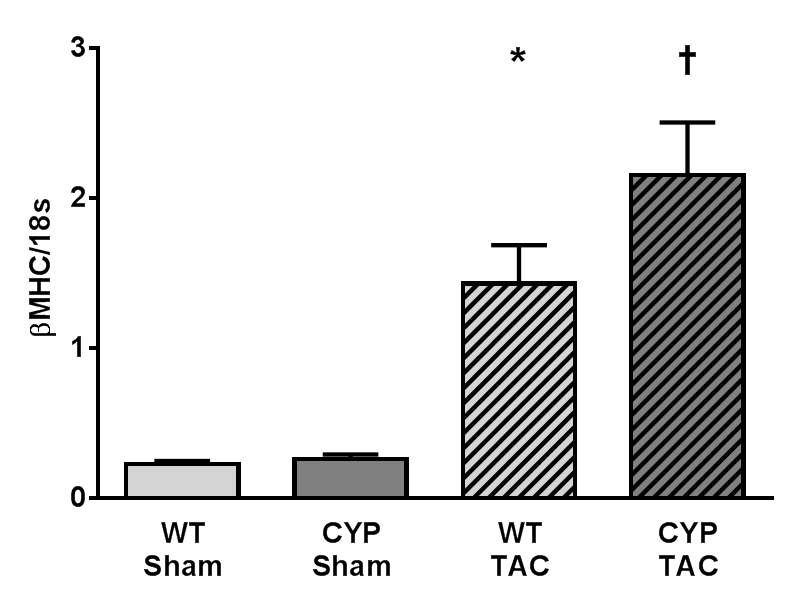
**

**Figure S1D**


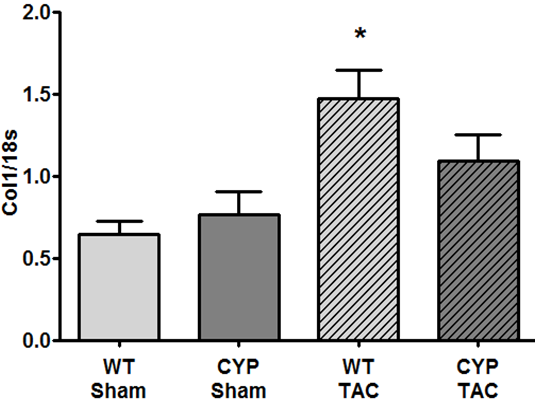


**Figure S1E**


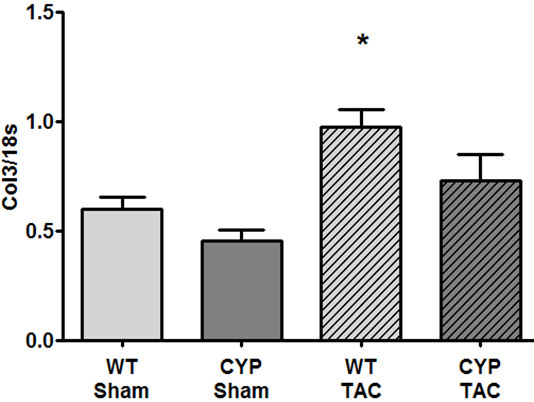


**Figure S1: Effect of chronic pressure overload on the expression of markers of hypertrophy and fibrosis in WT and CYP2J2-TG mice.** RNA isolated from left ventricular tissue 8 weeks after sham or TAC operation was reverse transcribed and analyzed by quantitative Taqman- or SYBR-PCR for the expression of ANP (A), BNP (B), βMHC (C) as well as Col1 (D) and Col3 (E). WT+Sham: n=13; CYP+Sham: n=7; WT+TAC: n=11; CYP+TAC: n=8. ANOVA, Post-Hoc Tukey; *p<0.05 vs. WT+Sham; ^†^p<0.05 vs. CYP+Sham.
