# Supplementary material for: CYP2J2 Overexpression Protects against Arrhythmia Susceptibility in Cardiac Hypertrophy
Source: PLoS One. 2013 Aug 30;8(8):e73490. doi: 10.1371/journal.pone.0073490 (PMC3758319; doi:10.1371/journal.pone.0073490)
Supplement: Figure S2 — Effect of chronic β-adrenergic stimulation on atrial fibrosis in WT and CYP2J2-TG mice. (DOCX) [file pone.0073490.s003.docx]

**Figure S2:**


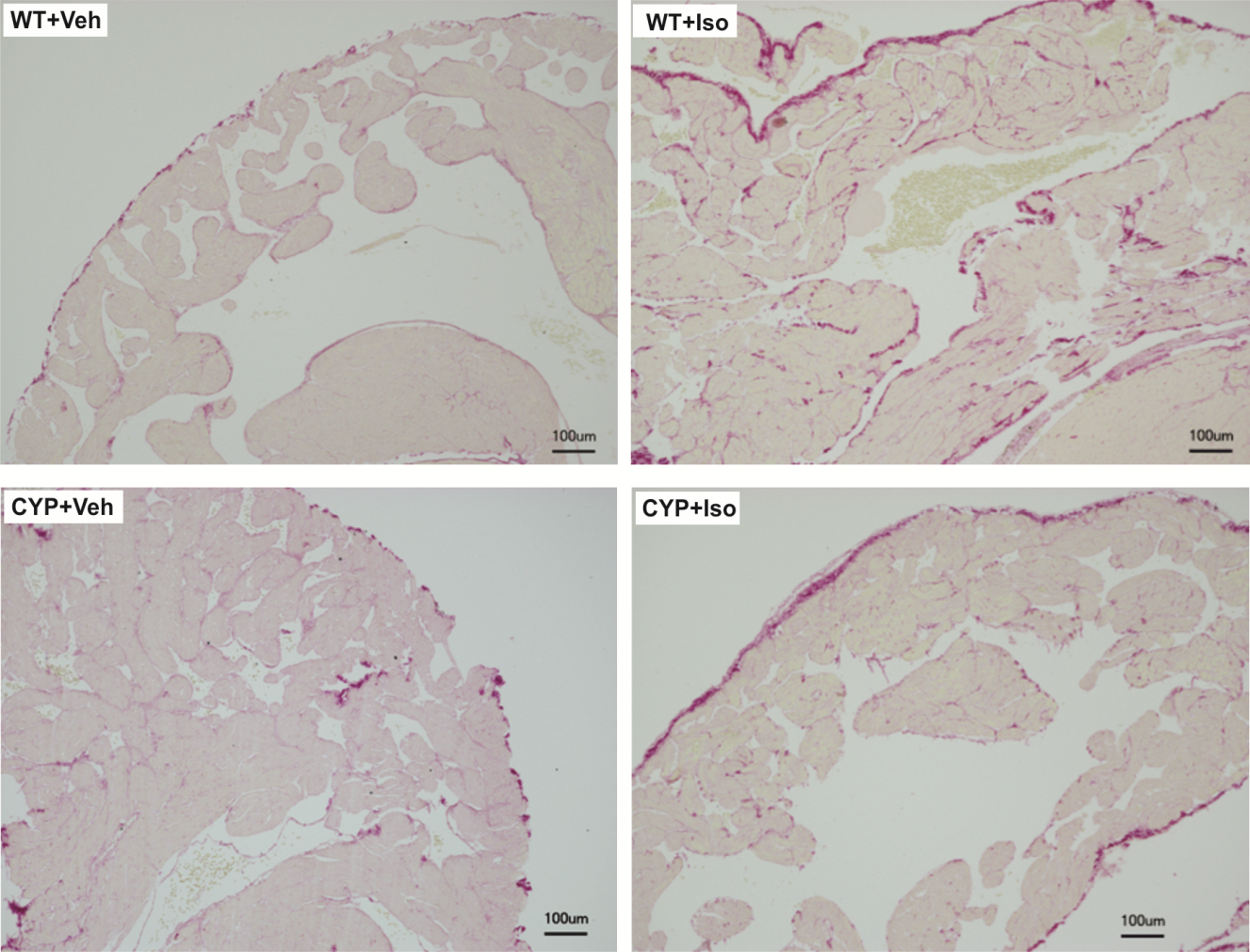


Figure S2: Effect of chronic β-adrenergic stimulation on atrial fibrosis in WT and CYP2J2-TG mice. Shown are representative examples of atrial slices from the different animal groups after Sirius red staining.
