# Supplementary material for: CYP2J2 Overexpression Protects against Arrhythmia Susceptibility in Cardiac Hypertrophy
Source: PLoS One. 2013 Aug 30;8(8):e73490. doi: 10.1371/journal.pone.0073490 (PMC3758319; doi:10.1371/journal.pone.0073490)
Supplement: Table S1 — Primer sequences for quantitative real-timeRT-PCR. (DOCX) [file pone.0073490.s004.docx]

Table S1: Primer sequences for quantitative real-time RT-PCR

ANP – Atrial natriuretic peptide; BNP – B-type natriuretic peptide; βMHC – β Myosin heavy chain; Col1 – Collagen 1; Col3 – Collagen 3.

| **Gene** | **Forward** | **Probe** | **Reverse** | **Size (bp)** |
| --- | --- | --- | --- | --- |
| 18s | ACATCCAAGGAAGGCAGCAG | FAM-CGCGCAAATTACCCACTCCCGAC-TAMRA | TTTTCGTCACTACCTCCCCG | 65 |
| ANP | AGGAGAAGATGCCGGTAGAAGA | FAM-AGGTCATGCCCCCGCAGGC-TAMRA | GCTTCCTCAGTCTGCTCACTCA | 89 |
| BNP | GCCAGTCTCCAGAGCAATTCA | - | GGGCCATTTCCTCCGACTT | 79 |
| βMHC | GCAAGGCCGAGGAGACGCAG | - | TGCCGGGACAGCTCCCCATT | 88 |
| Col1a2 | CTACTGGTGAAACCTGCATCCA | FAM-CCCAACCTGTAAACACCCCAGCGAAG-TAMRA | GGGCGCGGCTGTATGAG | 69 |
| Col3a1 | CTCACCCTTCTTCATCCCACTCTTA | - | ACATGGTTCTGGCTTCCAGACAT | 117 |
| Fibronectin | GGACCTGCAAACCTATAGCTGAGA | TGTTTTGATCATGCTGCTGGG | CTCCCCCACGACGTAGGA | 68 |
