# Supplementary material for: CYP2J2 Overexpression Protects against Arrhythmia Susceptibility in Cardiac Hypertrophy
Source: PLoS One. 2013 Aug 30;8(8):e73490. doi: 10.1371/journal.pone.0073490 (PMC3758319; doi:10.1371/journal.pone.0073490)
Supplement: Table S2 — Summary of echocardiographic data eight weeks after TAC. (DOCX) [file pone.0073490.s005.docx]

Table S2: Summary of echocardiographic data eight weeks after TAC.

WT – Wildtype; CYP – CYP2J2 overexpressing mice; TAC – Transverse aortic constriction; IVSd/s – End diastolic/systolic interventricular septal dimension; LVIDd/s – End diastolic/systolic left ventricular inner diameter; LVPWd/s – End diastolic/systolic left ventricular posterior wall dimension; LVM/TL – Left ventricular mass-to-tibia length; EF – Ejection fraction; FS –Fractional shortening.

|  | **WT Sham** | **CYP Sham** | **WT TAC** | **CYP TAC** |
| --- | --- | --- | --- | --- |
| **IVSd (mm)** | 0.69±0.01 | 0.70±0.02 | **1.01±0.03*** | **0.96±0.03^†^** |
| **LVIDd (mm)** | 4.66±0.04 | 4.38±0.11 | **5.38±0.11*** | **5.06±0.13^†^** |
| **LVPWd (mm)** | 0.63±0.01 | 0.66±0.03 | **0.99±0.03*** | **0.92±0.03^†^** |
| **IVSs (mm)** | 0.83±0.01 | 0.86±0.03 | **1.10±0.03*** | **1.06±0.03^†^** |
| **LVIDs (mm)** | 3.85±0.07 | 3.35±0.16 | **5.01±0.16*** | **4.48±0.20^†^** |
| **LVPWs (mm)** | 0.75±0.02 | 0.83±0.03 | **1.06±0.03*** | **1.04±0.03^†^** |
| **LVM/TL (mg/mm)** | 6.97±0.18 | 6.37±0.26 | 15.01±0.77 | 13.14±0.82 |
| **EF (%)** | 36.03±2.06 | 47.26±3.15 | 10.68±1.87 | 20.38±2.83 |
| **FS (%)** | 17.4±1.16 | **23.8±1.78*** | **4.56±0.88*** | **9.47±1.40^†^** |

p <0.05 * vs. WT Sham; † vs. CYP Sham; ‡ vs. WT TAC
