# Supplementary material for: CYP2J2 Overexpression Protects against Arrhythmia Susceptibility in Cardiac Hypertrophy
Source: PLoS One. 2013 Aug 30;8(8):e73490. doi: 10.1371/journal.pone.0073490 (PMC3758319; doi:10.1371/journal.pone.0073490)
Supplement: Table S3 — Summary of echocardiographic data after two weeks of β-adrenergic stimulation. (DOCX) [file pone.0073490.s006.docx]

**Table S3: Summary of echocardiographic data after two weeks of β-adrenergic stimulation.**

WT – Wildtype; CYP – CYP2J2 overexpressing mice; TAC – Transverse aortic constriction; IVSd/s – End diastolic/systolic interventricular septal dimension; LVIDd/s – End diastolic/systolic left ventricular inner diameter; LVPWd/s – End diastolic/systolic left ventricular posterior wall dimension; LVM/TL – Left ventricular mass-to-tibia length; EF – Ejection fraction; FS –Fractional shortening.

|  | **WT Vehicle** | **CYP Vehicle** | **WT Iso** | **CYP Iso** |
| --- | --- | --- | --- | --- |
| **IVSd (mm)** | 0.72±0,04 | 0.82±0.08 | **0.89±0.03*** | 0.92±0.05 |
| **LVIDd (mm)** | 4.24±0.10 | 4.07±0.11 | 4.60±0.11 | 4.28±0.09 |
| **LVPWd (mm)** | 0.72±0.04 | 0.81±0.07 | **0.88±0.03*** | 0.90±0.04 |
| **IVSs (mm)** | 1.06±0.07 | 1.22±0.14 | 1.23±0.05 | 1.33±0.06 |
| **LVIDs (mm)** | 3.17±0.13 | 2.93±0.10 | 3.53±0.15 | **3.01±0.10^‡^** |
| **LVPWs (mm)** | 1.05±0.05 | 1.19±0.10 | 1.20±0.06 | 1.28±0.06 |
| **LVM/TL (mg/mm)** | 6.91±0.26 | 6.93±0.15 | 9.99±0.55 | 9.89±0.37 |
| **EF (%)** | 51.13±3.24 | 57.30±1.73 | 47.41±3.22 | 59.02±1.70 |
| **FS (%)** | 25.3±1.65 | 28.19±1.10 | 23.54±1.79 | **29.78±1.37^‡^** |

p <0.05 * vs. WT Vehicle; † vs. CYP Vehicle; ‡ vs. WT Isoproterenol
